# Ectopic parathyroid and its role in surgical failure

**DOI:** 10.31744/einstein_journal/2025AI1403

**Published:** 2025-02-14

**Authors:** Murilo Catafesta das Neves, Marcello Rosano, Rodrigo Oliveira Santos

**Affiliations:** 1 Escola Paulista de Medicina Universidade Federal de São Paulo São Paulo SP Brazil Otorrinolaringologia e Cirurgia de Cabeça e Pescoço, Escola Paulista de Medicina, Universidade Federal de São Paulo, São Paulo, SP, Brazil.

**Figure 1:**
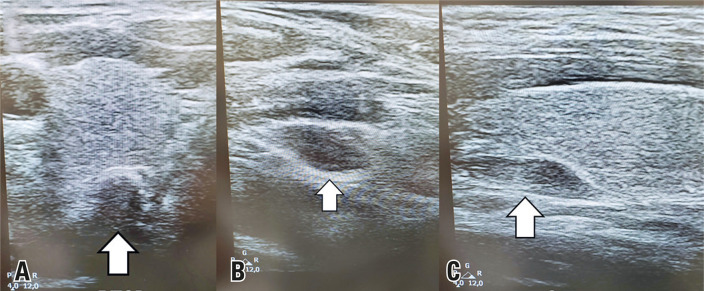
Preoperative ultrasound showing three parathyroid glands. (A) Arrow indicating the right superior parathyroid gland; (B) arrow indicating the right inferior parathyroid gland; (C) arrow indicating the left superior parathyroid gland

**Figure 2:**
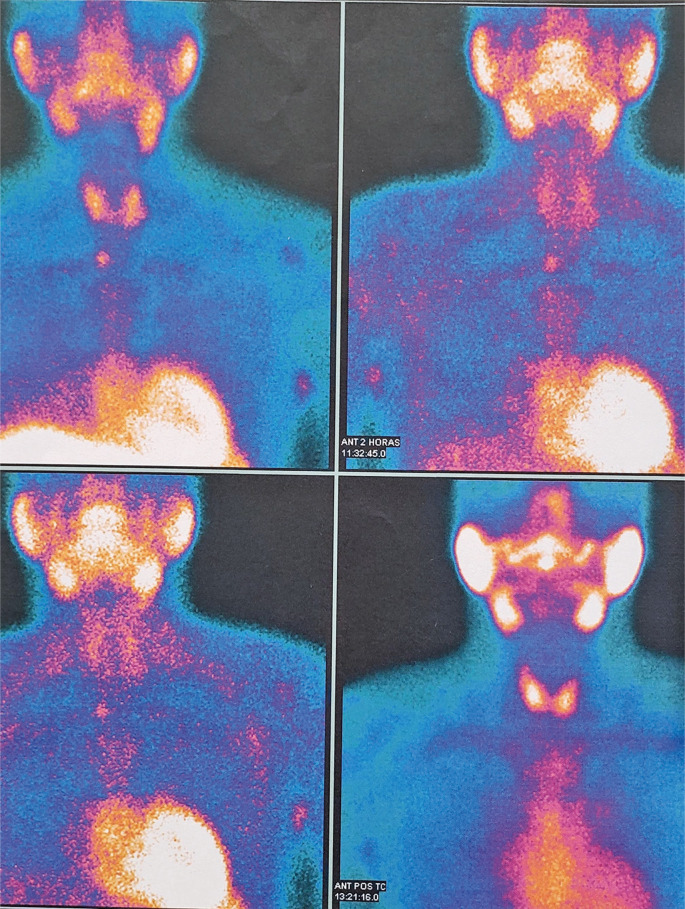
Planar Sestamibi scan showing an inferior parathyroid gland located in the upper right mediastinum

**Figure 3:**
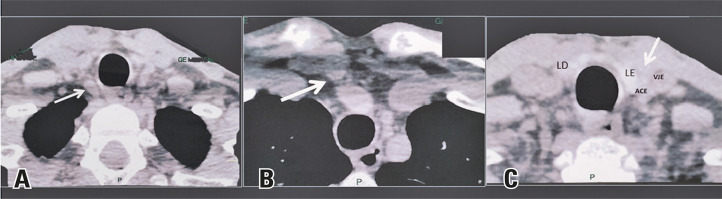
Preoperative SPECT-CT showing three possible parathyroid glands corroborating the ultrasound findings. The white arrows indicate the right superior parathyroid gland (A), right inferior parathyroid gland (B), and left superior parathyroid gland (C)

A 32-year-old man with renal hyperparathyroidism was referred for surgical treatment after exhausting clinical measures for metabolic control.^1,2^Combined analysis of neck ultrasound (Figure 1) and Sestamibi (Figure 2)/SPECT-CT (Figure 3) findings enabled the identification of three possible enlarged parathyroid glands.^3^The right and left superior parathyroid glands were located in their usual positions beneath the thyroid gland, while the right inferior parathyroid gland was found in the upper mediastinum.

During surgery, the three identified glands were successfully located and removed. However, despite extensive dissection of the neck and upper mediastinum, the left inferior parathyroid gland could not be found.^4^ The intraoperative PTH failed to decline as expected, indicating the presence of a fourth ectopic gland.^5^

The patient’s condition showed mild metabolic improvement, but elevated PTH levels persisted, associated with hypercalcemia.

**Figure 4:**
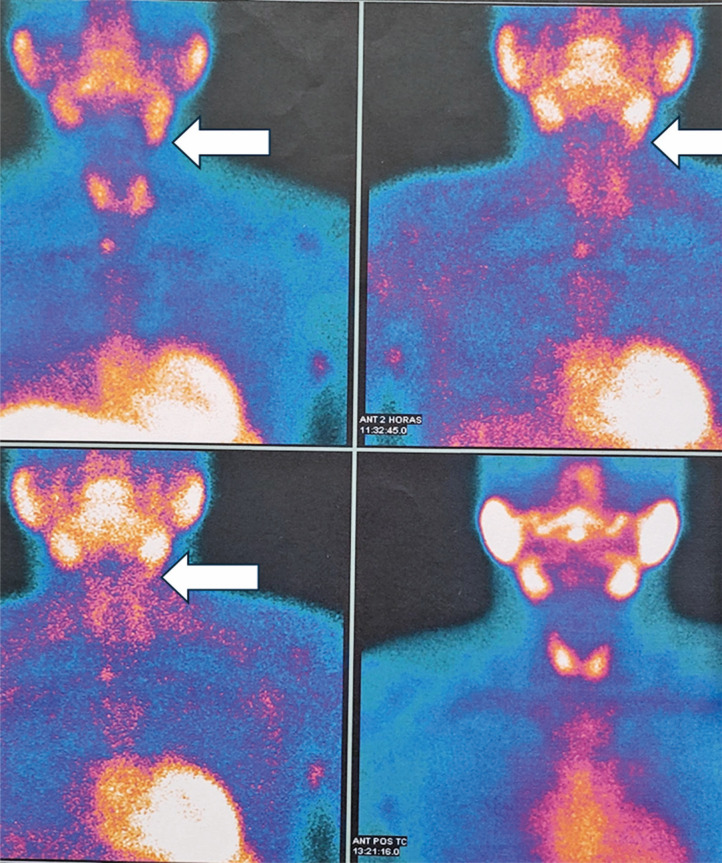
Reevaluation of Sestamibi imaging showing asymmetric uptake along the left submandibular region (indicated by the white arrows)

**Figure 5:**
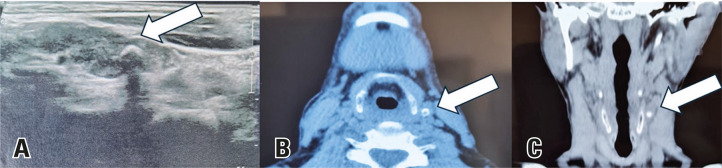
Target ultrasound (A) and computed tomography (B and C) showing an ectopic, non-descended inferior left parathyroid gland located in the submandibular region. The white arrows indicate the ectopic gland. Identification was facilitated by the observation of an intraparenchymal calcification

A comprehensive review of the Sestamibi/SPECT-CT images suggested the possibility of an undescended left parathyroid gland adjacent to the submandibular gland (Figure 4). Targeted ultrasound and CT (Figure 5), along with fine-needle aspiration, confirmed the presence of an ectopic, non-descended inferior left parathyroid gland, which was subsequently excised during a second surgery via a direct approach to the submandibular area.

It is essential to thoroughly evaluate localization exams to consider all potential ectopic locations of parathyroid glands and minimize the risk of surgical failure. Although undescended parathyroid glands are rare, ectopic glands are a significant cause of initial surgical failure and reoperation.^6^
